# Easyworm: an open-source software tool to determine the mechanical properties of worm-like chains

**DOI:** 10.1186/1751-0473-9-16

**Published:** 2014-07-10

**Authors:** Guillaume Lamour, Julius B Kirkegaard, Hongbin Li, Tuomas PJ Knowles, Jörg Gsponer

**Affiliations:** 1 Centre for High-Throughput Biology, University of British Colombia, Vancouver, BC V6T 1Z4, Canada; 2 Department of Chemistry, University of British Columbia, Vancouver, BC V6T 1Z1, Canada; 3 Department of Biochemistry & Molecular Biology, University of British Colombia, Vancouver, BC V6T 2A1, Canada; 4 Department of Chemistry, University of Cambridge, Cambridge CB2 1EW, UK

**Keywords:** Matlab, GUI, Polymer, Worm-like chain model, Persistence length, Young’s modulus, AFM

## Abstract

**Background:**

A growing spectrum of applications for natural and synthetic polymers, whether in industry or for biomedical research, demands for fast and universally applicable tools to determine the mechanical properties of very diverse polymers. To date, determining these properties is the privilege of a limited circle of biophysicists and engineers with appropriate technical skills.

**Findings:**

Easyworm is a user-friendly software suite coded in MATLAB that simplifies the image analysis of individual polymeric chains and the extraction of the mechanical properties of these chains. Easyworm contains a comprehensive set of tools that, amongst others, allow the persistence length of single chains and the Young’s modulus of elasticity to be calculated in multiple ways from images of polymers obtained by a variety of techniques (*e.g.* atomic force microscopy, electron, contrast-phase, or epifluorescence microscopy).

**Conclusions:**

Easyworm thus provides a simple and efficient tool for specialists and non-specialists alike to solve a common problem in (bio)polymer science. Stand-alone executables and shell scripts are provided along with source code for further development.

## Introduction

Although different approaches have been developed over the years to determine the nanomechanical properties of different biopolymers [1-3], it is mainly biophysicists and engineers with appropriate technical skills who have been able to use them. However, the growing number of technological applications for functional biopolymers such as modified cytoskeletal filaments or engineered DNA [4,5] asks for a fast and easy way to determine their mechanical properties that is also accessible to non-specialists. Here we present a new software tool, Easyworm [6], for the determination of the persistence length of polymer chains and derivation of their axial elastic modulus. This open-source software provides accurate measurements of the persistence length varied over 6 orders of magnitude (from nm to mm ranges) and can be used by specialists and non-specialists alike.

## Implementation

Easyworm consists of several graphical user interfaces (GUI) functioning as stand-alone applications for Microsoft Windows or Linux operating systems. They require the appropriate MATLAB Compiler Runtime (MCR) version to be installed. Source code (.m) files along with GUIDE .fig files will also work under a MATLAB environment. They can also be deployed as stand-alone executables or shell scripts, providing the MATLAB compiler toolbox is installed on the development machine. MCR versions, executable files, shell scripts and the source code are freely available at http://www.chibi.ubc.ca/faculty/joerg-gsponer/gsponer-lab/software/easyworm. Detailed installation notes are provided on the same webpage. In addition, step-by-step instructions of how to use the software are provided in the Additional file 1 of this paper (Easyworm_SuppInfo.pdf).

## Methods overview

**Figure 1:**
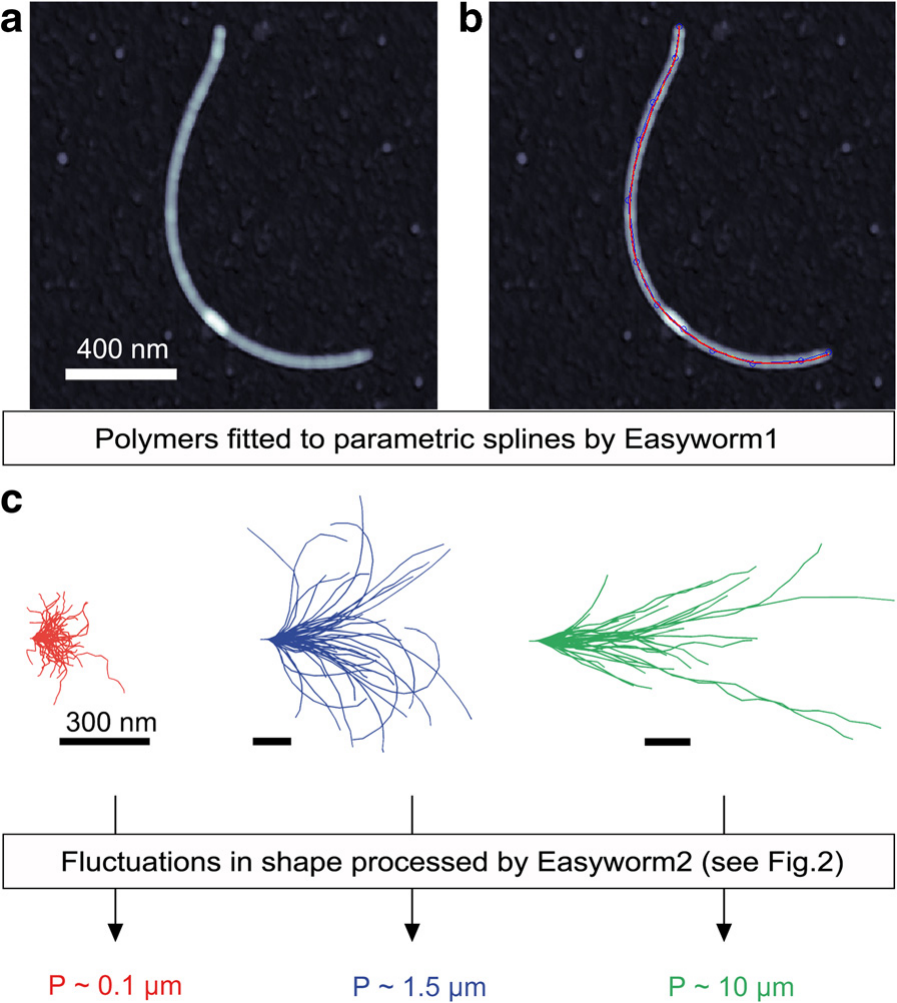
**Easyworm workflow. (a)** Atomic force microscopy image of an amyloid fibril. **(b)** Same image as in **(a)**, in which the contour of the fibril has been fitted to a parametric spline (red line; see Additional file 1: Note S1). **(c)** Three distinct amyloid fibril samples plotted with their initial tangents aligned to facilitate visualization. P is the persistence length of the fibrils, derived from the measures shown in Figure 2.

Easyworm is optimized for analyzing images of individual polymer chains taken by atomic force microscopy (AFM; Figure 1) but can also be used for analyzing images taken by other methods (*e.g.* electron microscopy, epifluorescence, or simple contrast-phase optical microscopy). Minimal user input is required in order to fit the contour of polymers to parametric splines (see Figure 1b) after uploading height maps in the first GUI, *Easyworm1* (for detailed instructions see Additional file 1: Figure S1 and Note S1 in Easyworm_SuppInfo.pdf). Then *Easyworm2* (second GUI; Additional file 1: Figure S2 and Note S2) is used to derive the mechanical properties from the data collected by *Easyworm1*.

### Persistence length calculations

**Figure 2:**
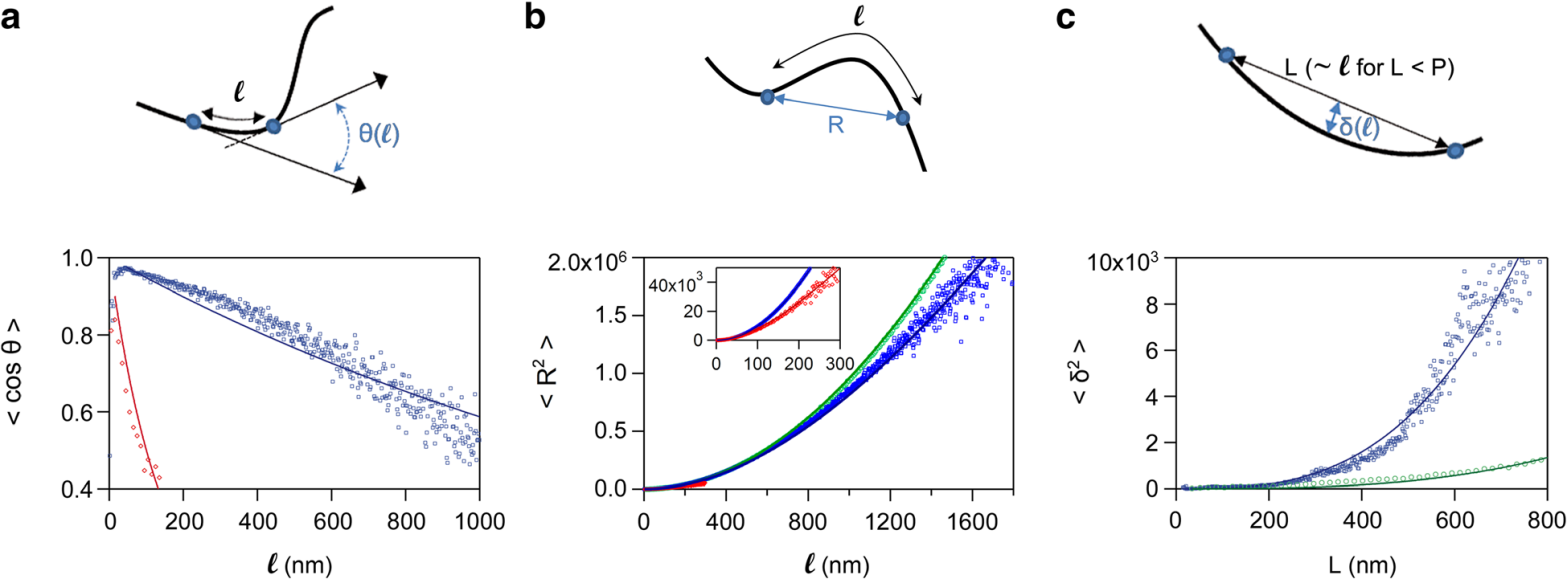
**Three distinct measures used to calculate the persistence length.** The data were generated from the fibrils plotted in Figure 1. **(a)** cos θ, **(b)** mean square of the end-to-end distances R, and **(c)** mean square of the deviations δ to secant midpoints, as a function of *ℓ* (see main text for details). Red diamonds, blue squares, and green circles represent data for three different amyloid fibrils. Lines: fits of the worm-like chain model to the data (according to the equations indicated in the main text). Persistence lengths (P) derived from the fits are indicated in Figure 1 (same color as the fitted curves).

The persistence length P of a sample of individual polymeric chains can be obtained *via* three distinct measures all derived from the worm-like chain model (WLC) for semi-flexible polymers. The choice of the measure to calculate P is highly dependent on the value of P with regard to the contour length of the polymer. For instance, P can be much higher (*e.g.* microtubules) or much lower (*e.g.* DNA) than the contour length. For quite flexible polymers, it is recommended to monitor the decay of tangent-tangent correlations (Figure 2a) according to [1]:

(1) $$<\cos\;\theta >\;=\;e^{-\;\frac{\ell }{\text{sP}}}$$

where θ is the angle between two segments of the spline separated by a distance *ℓ* along the chain contour. s is a surface parameter that is set by the user to a value of 2 for chains that have equilibrated on the 2D surface or to a value of 1.5 ± 0.5 for nonequilibrated chains (see Easyworm_SuppInfo.pdf for more details). Another available option [3] (Figure 2b) is the measurement of the mean square of the end-to-end distance R as a function of *ℓ*:

(2) $$<R^{2}>\;=\;2\text{sP}\ell \;\left(1-\frac{\text{sP}}{\ell }\left(1-e^{-\;\frac{\ell }{\text{sP}}}\right)\right)$$

If the contour length of the fibrils is much lower than their persistence length, the user can choose another measure [2] to derive P (Figure 2c):

(3) $$<\;\delta^{2}>\;=\;\frac{L^{3}\;}{24\text{sP}}$$

where δ is the deviation from the chain to the midpoint of a secant of length L joining two knots of the spline for each combination of knots over the chain contour. The fluctuation expressed in Eq. 3 is valid only for L < < P. In addition, L can be assimilated to *ℓ* (as defined in Eqs. 1 and 2) for values of L lower than the persistence length of the chain. All the functions described in Eqs. 1–3 assume that the chains are not self-avoiding.

### Uncertainties on persistence length calculations

Uncertainties in the calculated persistence lengths are determined *via* random resampling using the standard method of bootstrap with replacement [7]. In short, new chain samples (bootstrap samples) that contain k chains are randomly chosen from the available k chains. As the bootstrap samples are different from the original sample, any chain can be selected more than once (see Ref [7] for details). For each bootstrap sample < cos θ >, < R^2^ >, or < δ^2^ > values are binned at regular length intervals as in Figure 2. Different forms of the WLC model are then fitted to the data. *n* (default 10) bootstrapping operations are done, and the mean of the *n* values returned at each iteration is the persistence length of the polymer. The standard deviation on the *n* values is the uncertainty on P (to which the uncertainty on the fractional dimension is propagated when considering non-equilibrated polymers, see Additional file 1: Methods).

### Additional tools

A complementary set of tools is provided in several graphical user interfaces that serve detailed analyses of the data, including the plotting of polymers (Figure 1c) and the statistical treatment of polymer contour lengths (see Additional file 1: Figure S2 and Note S2). For instance, the user can plot a histogram of the distribution of polymer contour lengths, and Gaussian fitting of the distribution can be done within the GUI. Also available is the possibility to derive an axial elastic modulus from three distinct models for the cross-sectional geometry of the polymer. Importantly, multiple control functions are included. First, the ability to adapt the fitting of the chain contour by setting a user-defined "fitting parameter" (see Additional file 1: Figure S1 and Note S1). In practice, this allows preserving the accuracy of the measurements at any given resolution providing it meets minimum requirements (see Additional file 1: Note S1 for details). Second, two independent tests [3,8] to determine whether or not the polymers have fully equilibrated in 2D, which can influence the choice of the model used to be fitted to data (see next section, where these two tests are described in detail). Third, a Monte-Carlo-based method described previously [3] was implemented into another graphical user interface (*Synchains*) to generate *in silico* polymers with user-defined persistence lengths (Additional file 1: Figure S3 and Note S3). In short, if P is the persistence length, then the small angles θ between discrete segments located at a distance *ℓ* apart have a probability density P:

(4) $$P{\left(\theta \left(\ell \right)\right)}_{2D}\;\alpha \;e^{-\frac{P\theta^{2}}{2\ell }}$$

The standard deviation of this normal distribution is $<\theta^{2}{(\ell )>}_{2D}\;=\;\sqrt{\ell /P}$. Therefore, we generated *n* segments of length *ℓ* joined at each other’s ends and forming angles *θ* randomly chosen according to a normal distribution around a mean 0 and with a standard deviation equal to $\sqrt{\ell /P}$. Such synthetic chains are illustrated in Additional file 1: Figure S4. Refer to Additional file 1: Note S4 for details on how synthetic chains were used in the different analyses contained in this study.

### Equilibration on the 2D surface

**Figure 3:**
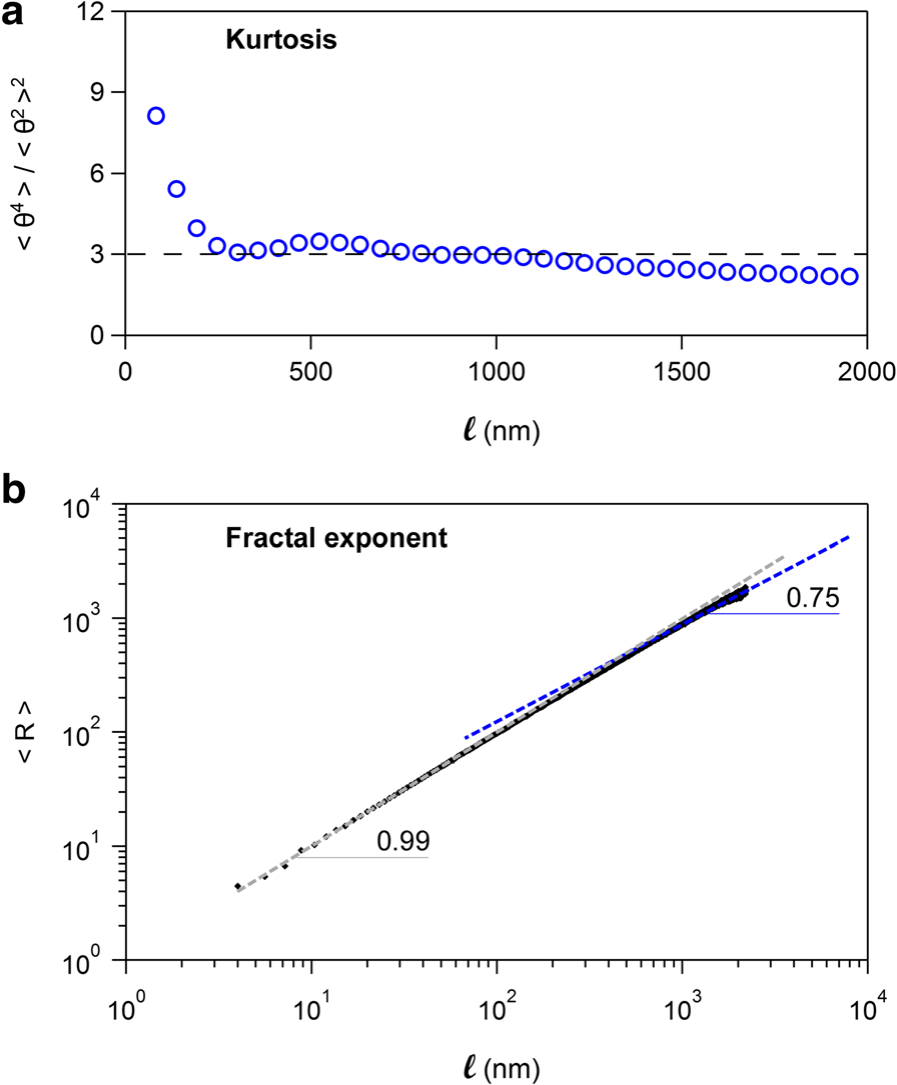
**Two independent tests to determine whether the polymers have fully equilibrated in 2 dimensions. (a)** Kurtosis of the θ distribution as a function of *ℓ* (blue circles). θ is the angle formed by two discrete chain segments separated by a distance *ℓ* along the chain contour. A kurtosis equal to 3 (broken line) indicates that the polymers have fully equilibrated on the 2D (see also Figure 4). **(b)** Mean end-to-end distance R as a function of *ℓ*. For *ℓ* > P where P is the persistence length, a slope of 0.75 indicates full equilibration in 2D. The data displayed in **(a)** and **(b)** were collected for amyloid fibrils seeded on glass, where full equilibration in 2D is expected [9].

**Figure 4:**
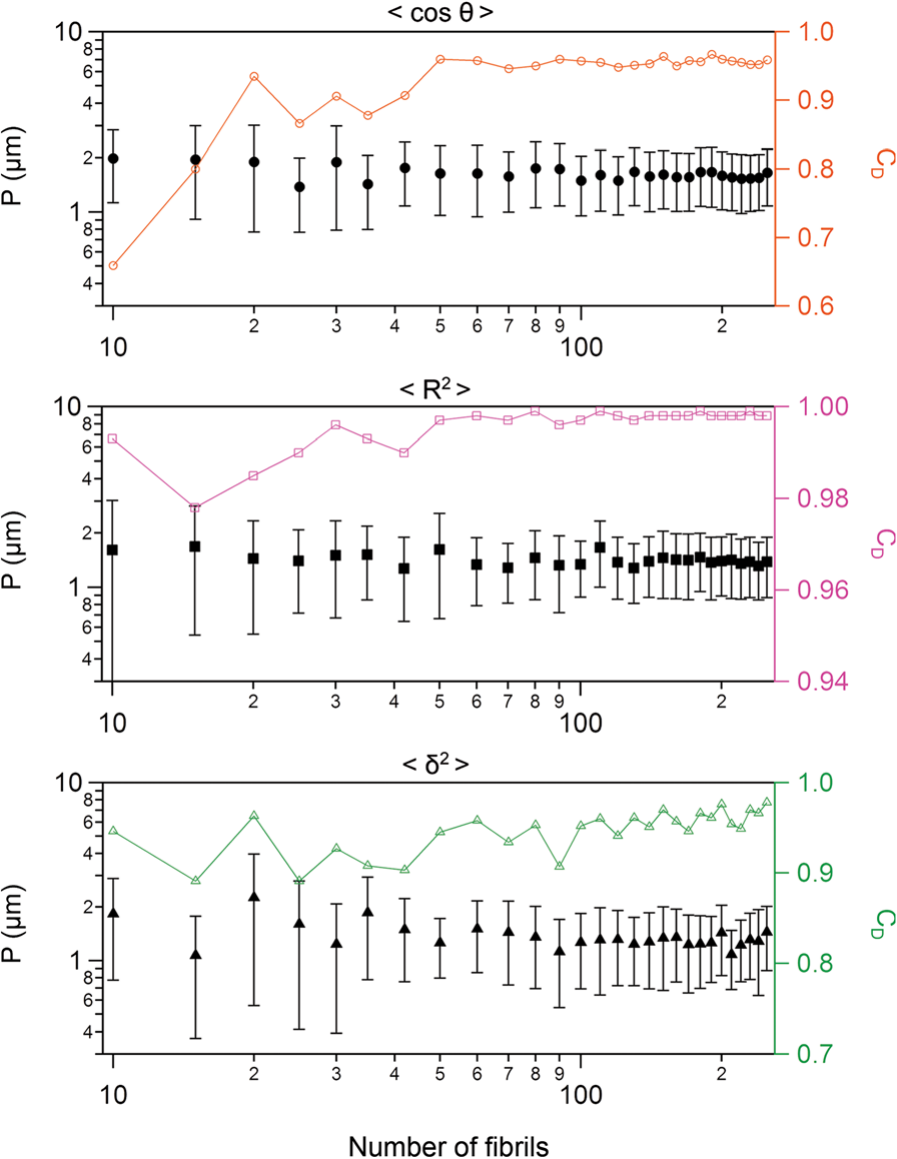
**Precision of persistence length measurements by Easyworm.** Persistence length P (of W sample, see Additional file 1: Table S1) is displayed as a function of the number of chains used to perform the analysis (black symbols). The coefficient of determination C_D_ associated with each fit realized is indicated in colored open symbols and reveals how well the data fit the model. The contour length of the chains analyzed here is ~1.0 ± 0.5 μm (mean ± SD). All data points and their associated error bars are the result of 10 bootstrapping operations (see main text for details). Refer to main text for the meaning of cos θ, R, and δ.

*Easyworm2* contains two functions that can help to determine whether or not the chains fully equilibrate in 2D (Figure 3). The first one calculates the ratio of the even moments, *i.e.* the kurtosis of the distribution of the θ angle (Figure 3a). If the chains fully equilibrate in 2D, then the θ distribution is Gaussian [3], and in the range where angles θ are still fully correlated (*i.e.*, *ℓ* ≤ P and < cos θ > ≥ 0.6, see Figure 4), the kurtosis results in:

(5) $$\frac{<\theta^{4}\left(\ell \right){>}_{2D}}{<\theta^{2}\left(\ell \right){>}_{2D}^{2}}\;=\;3$$

For distances *ℓ* greater than P, the kurtosis does not equal to 3 anymore and starts decreasing. When the θ angles become completely uncorrelated (*i.e.*, < cos θ > = 0), then the distribution of θ is uniform, that is, all θ angles are equiprobable. Only when this condition is fully met the kurtosis equals 1.8 (see Additional file 1: Figure S4 for more details).

Another function implemented in Easyworm allows for the fast determination of the slope of < R > as a function of *ℓ* on any given range of *ℓ* (Figure 3b). Provided the contour length interval defined by the user to calculate this slope (corresponding to a scaling or fractal exponent [8]) is located above the persistence length (*i.e.* for *ℓ* > P), the slope is equal to 0.75 for a self-avoiding random walk in 2D [8]. We note that for our software, in practice, this measurement is accurate only for contour length values comprised between P and ~3P, since above 3P the number of data points available are usually too low to produce a measurement that is statistically significant.

## Results and performance evaluation

**Figure 5:**
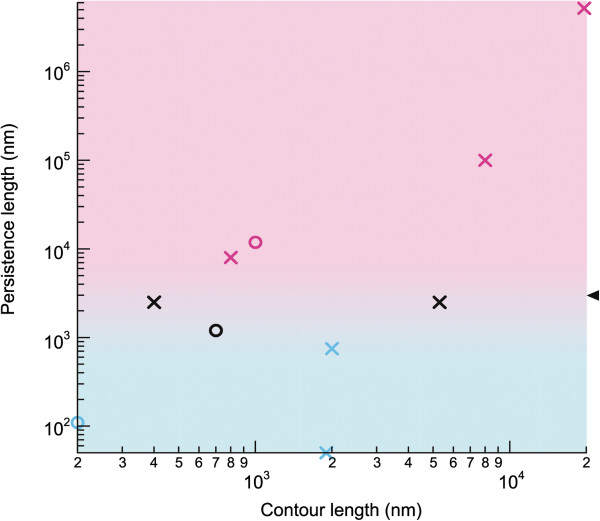
**Graphical guide indicating which measure should be used to derive the persistence length.** The crosses (synthetic chains of known persistence length) and circles (experimental polymers) correspond to data points that are given in Table 1 and Additional file 1: Table S1. Light blue markers represent the samples for which the most reliable calculations of persistence length are achieved by measuring < cos θ > and/or < R^2^ >, whereas purple markers indicate samples for which measuring < δ^2^ > provides the best estimation of the persistence length. Black markers indicate samples for which all measures provided reliable results. Therefore, the light blue region indicates where measures of < cos θ > and/or < R^2^ > should be used to provide the most reliable value for the persistence length, whereas the pink region indicates where measure of < δ^2^ > should be used. Note that background coloring serves as a guide only and that the frontier between light blue and pink regions (indicated by the black arrowhead) is strongly correlated to the image size available for analysis (typically, in the orders of 1–10 μm).

We used *in silico* polymers (see *Additional tools* section) in order to test the accuracy of the measurements made by Easyworm. The benchmarks (see Table 1) indicate that Easyworm is able to provide reliable results over a very wide range of persistence lengths P from that of DNA (P ≈ 50 nm [3]) to that of microtubules (P ≈ 5.2 mm [2]). In another test performed on amyloid fibrils generated *in vitro*, we determined that relatively good precision on the measurements of P can be obtained with a minimum of 50–60 chains that have contour length C_L_ ~1.0 ± 0.5 nm (Figure 4). The number of chains required will be higher if C_L_ is lower. As Easyworm can be used to derive persistence lengths varying over several orders of magnitude, we included a graphical guide that provides the user with indications on which measure to use depending on the persistence length of the sample (Figure 5). For instance, when considering fibrils having P > 5 μm, monitoring the end-to-end distance R along the polymer contour is not as efficient as monitoring the deviations δ from the polymer to secant midpoints (see Table 1).

**Table 1:** Evaluation of the measurement accuracy using synthetic polymers with known persistence lengths as test samples

^*^Sample	**N chains**	**Persistence length according to all 3 measures (nm) **^**†**^**(C**_**D**_**) **^**‡**^**[interval; nm]**		
		^▲^**< R**^**2**^ **> = *f *** **(*ℓ*)**	^▲^**< cos θ > = *f *** **(*ℓ*)**	^▲^**< δ**^**2**^** > = *f *** **(*ℓ*)**
^§^SP50	38	^§^68 ± 3 (0.996)	^§^70 ± 6 (0.927)	–
		[0; 500]	[20; 500]	
SP750	78	777 ± 114 (0.999)	728 ± 32 (0.968)	538 ± 28 (0.961)
		[0; 1900]	[50; 1000]	[0; 300]
^¶^SP2500-1	44	2867 ± 372 (0.999)	2599 ± 506 (0.947)	2986 ± 914 (0.923)
		[0; 600]	[20; 500]	[0; 600]
^¶^SP2500-2	35	3047 ± 496 (0.999)	3015 ± 590 (0.966)	2894 ± 608 (0.991)
		[0; 2500]	[40; 2500]	[0; 1200]
^¤^SP2500-2	35	2525 ± 191 (0.999)	2542 ± 214 (0.960)	2441 ± 318 (0.966)
		[0; 600]	[40; 600]	[0; 600]
SP8000	41	7280 ± 1060 (0.999)	6669 ± 494 (0.789)	8262 ± 1083 (0.949)
		[0; 1200]	[20; 700]	[0; 800]
SP1e5	48	64264 ± 5514 (0.999)	–	86475 ± 14480 (0.985)
		[0; 3500]		[0; 3500]
SP5.2e6	70	1.49e5 ± 0.13e5 (0.999)	–	5.64e6 ± 0.85e6 (0.994)
		[0; 19500]		[0; 18000]

Refer to Additional file 1: Note S4 and Table S1 for details on how the data in this table was generated with *Synchains* and analyzed with *Easyworm*.

^*^Each number in the sample names corresponds to the persistence length P (in nm) that was used to generate one particular synthetic polymer (SP), *e.g.* for SP50, P was set to 50 nm.

^†^C_D_ is the coefficient of determination (usually noted "R^2^" but not here because R is already used to refer to the end-to-end distance).

^‡^[interval] is the range of distance *ℓ* (along the chain contour) on which each fit was made.

^▲^Mean square of the end-to-end distance R, tangent-tangent correlations < cos θ > and mean square of the deviations δ to secant midpoint, as described in Eqs. 1–3.

^§^We excluded chains displaying non-self-avoiding random walk from the analysis of SP50 chains (see Additional file 1: Figure S5). Therefore the value of 70 nm for P was expected [3].

^¶^SP2500-1 and SP2500-2 differ by their contour length (respectively 0.4 ± 0.2 and 5.3 ± 2.8 μm).

^¤^The calculated value for P is closer to the theoretical value than in the same sample in the above line. This is probably due a larger amount of data available for the shortest *ℓ* distances, hence rendering statistical analysis more reliable.

## Conclusions

Easyworm is a tool for researchers in need of a fast and ready-to-use program in order to determine the persistence length and derive the elastic modulus of their polymers, whether these are amyloid fibrils [9] or any nano- or micro-filaments. In addition to determining the mechanical properties, Easyworm also provides complementary tools to analyze polymer contour lengths, create synthetic polymers, visualize polymers and generate output files for plotting purposes.

## Competing interests

The authors declare no competing interests.

## Authors’ contributions

GL developed the software from TPJK’s initial code. GL and JBK tested the software. HBL and JG advised on the methods. GL wrote the manuscript. All authors commented and edited the manuscript. All authors have read and approved the final manuscript.

## Authors’ information

GL is a postdoctoral research fellow in the laboratories of JG and HBL at the University of British Columbia (Canada). JBK is a student in TPJK’s laboratory at the University of Cambridge (UK). HBL is an associate professor in Chemistry, TPJK a lecturer in Physical Chemistry, and JG an assistant professor in Biochemistry.

## Supplementary Material

Additional file 1 **(Easyworm_SuppInfo.pdf) is available with the online version of this article.** It contains Additional file Methods, Table S1, Figures S1-S5, Notes S1-S4 (including step-by-step instructions to use the software), and a list of References. Click here for file
