# Supplementary material for: Easyworm: an open-source software tool to determine the mechanical properties of worm-like chains
Source: Source Code Biol Med. 2014 Jul 10;9:16. doi: 10.1186/1751-0473-9-16 (PMC4106204; doi:10.1186/1751-0473-9-16)
Supplement: Additional file 1 — (Easyworm_SuppInfo.pdf) is available with the online version of this article. It contains Additional file Methods, Table S1, Figures S1-S5, Notes S1-S4 (including step-by-step instructions to use the software), and a list of References. [file 1751-0473-9-16-S1.pdf]

Supplementary Information for:

Easyworm: an open-source software tool to determine the mechanical properties of worm-like chains

Guillaume Lamour, Julius B. Kirkegaard, Hongbin Li, Tuomas P. J. Knowles, and Jörg Gsponer

Contents

Supplementary Methods

Equilibration or nonequilibration behavior on the 2D surface

Calculation of the second moment of area and derivation of the elastic modulus

Table S1

Figures S1–S5

Notes S1–S4

(Step-by-step instructions to use Easyworm GUIs can be found in Notes S1–S3)

Supplementary Methods

Equilibration or nonequilibration behavior on the 2D surface

In Eqs 1–3 (see main text), s is a parameter reflecting the influence of the surface-free energy of the substrate and has a value between 1 and 2. If we consider that the chains fully equilibrate on the substrate, that is, in 2 dimensions, then s = 2. In the case of a polymer equilibrated in 3 dimensions, s = 1 and the persistence length is exactly twice as much as P derived from the equations where s = 2 [1]. If we consider that chains are kinetically trapped in non-equilibrated conformations (case of strong interaction between polymer chains and substrate), then we can make the simple approximation that the fluctuations of the polymer are in between 2D and 3D states, and calculate the midpoint fluctuations using s = 1.5, as in previous studies [2, 3]. Then, the fractional dimension is 2.5 ± 0.5, and the uncertainty on this parameter is propagated to the final error estimate.

Calculation of the second moment of area and derivation of the elastic modulus

Once P has been determined, *Easyworm2* provides additional convenient tools that can help to determine the axial elastic modulus E of the polymeric chain under consideration (*e.g.* amyloid or collagen fibrils), by using the formula:

$$E = P.k_B.T / I, \qquad \text{(Eq. S1)}$$

where T is the room temperature, k_B the Boltzmann constant and I the cross-sectional second moment of area. I is a measure of the intrinsic resistance to bending. It is expressed in m^4 and varies in function of both the cross-sectional area and the geometry of the cross-section. Depending on whether the user has measurements on the height (h) alone or on both the height and width (w) of the chain under consideration, different models can be used in *Easyworm2* to calculate I. The three distinct models to calculate I are (with distinct geometries of the cross-sectional area in parentheses): helical/cylindrical (*circular;* I_C), ellipsoidal (*ellipsoidal;* I_E), and tape/ribbon-like (*rectangular;* I_R), respectively. The following expressions are used to calculate I as well as I_- and I_+ for each of the three models:

$$I_C = \frac{\pi.h^4}{64}; \quad I_{C-} = \frac{\pi.(\langle h\rangle - \Delta h)^4}{64.\zeta}; \quad I_{C+} = \frac{\pi.(\langle h\rangle + \Delta h)^4}{64}; \qquad \text{(Eq. S2)}$$

$$I_E = \frac{\pi.w.h^3}{64}; \quad I_{E-} = \frac{\pi.(\langle w\rangle - \Delta w).(\langle h\rangle - \Delta h)^3}{64.\zeta}; \quad I_{E+} = \frac{\pi.(\langle w\rangle + \Delta w).(\langle h\rangle + \Delta h)^3}{64}; \qquad \text{(Eq. S3)}$$

$$I_R = \frac{w.h^3}{12}; \quad I_{R-} = \frac{(\langle w\rangle - \Delta w).(\langle h\rangle - \Delta h)^3}{12.\zeta}; \quad I_{R+} = \frac{(\langle w\rangle + \Delta w).(\langle h\rangle + \Delta h)^3}{12}. \qquad \text{(Eq. S4)}$$

In these equations, ζ is a parameter calculated from the parallel axis theorem and which is related to the effect (on I) of offset $1 - r_n$ from the centre of the filaments, where r_n is the radius of one of the n tightly

packed protofilaments inside a unit circle (equivalent to fibril cross-sectional perimeter). ζ was taken (equal to 2.66) for a previous study [4] that determined that this number could be applied to the experimentally known range of 6 protofilaments in the mature fibril. Δh and Δw are the uncertainties on the height and on the width of the polymer, respectively. Precisely, the Young's moduli for each models are obtained using $E_{-} = B_{R^-}/I_{+}$, $E = B_R/I$, and $E_{+} = B_{R^+}/L$, where $B_R = P.k_B.T$ is the bending rigidity.

Table S1: Details of the thermal fluctuations analysis for each experimental and synthetic fibril samples.

Sample	Substrate	*N chains (∑contour, µm)	Measured chain length, µm (Max. value, µm)	†Knots intervals, nm	‡Data points
▲W	mica	253 (241.7)	1.0 ± 0.5 (2.8)	26 ± 5	254,341
▲NT	mica	44 (43.0)	1.0 ± 0.7 (3.4)	26 ± 9	49,835
▲FV	mica	198 (31.4)	0.2 ± 0.1 (0.4)	15 ± 4	13,222
§SP50	–	38 (71.2)	1.9 ± 0.3 (3.0)	20 ± 4	191,047
§SP750	–	78 (151.8)	2.0 ± 0.7 (3.6)	25 ± 4	260,857
§SP2500-1	–	44 (18.6)	0.4 ± 0.2 (0.8)	30 ± 4	5,136
§SP2500-2	–	35 (115.1)	5.3 ± 2.8 (5.3)	35 ± 8	193,444
§SP8000	–	41 (31.6)	0.8 ± 0.4 (1.7)	24 ± 5	23,869
§SP1e5	–	48 (383.5)	8.0 ± 0.3 (8.8)	50 ± 1	614,475
§SP5.2e6	–	70 (1371.5)	19.6 ± 0.2 (20.1)	64 ± 1	3 308,503

*"∑contour" means the sum of the contour length of all of the N chains analysed per sample.

†The average distance between spline knots is designated by "Knots interval".

‡ "Data points" corresponds to the total number of points available for statistical analysis, *i.e.* those that were binned and used for fitting.

▲Fibril samples W, NT and FV were all prepared from mouse prion protein PrP23-231 and imaged by AC mode atomic force microscopy in ambient air [3]. The "W" letter refers to the wild-type construct. Abbreviated letters "NT", and "FV" refer to the mutant constructs S170N-N174T (NT) and L108F-T189V (FV). The persistence lengths of W, NT, and FV samples are displayed in Fig. 1 (~1.5, ~10, and ~0.1 µm, respectively).

§Samples whose names start by SP are synthetic polymers generated *in silico* with *Synchains*.

Figure S1:

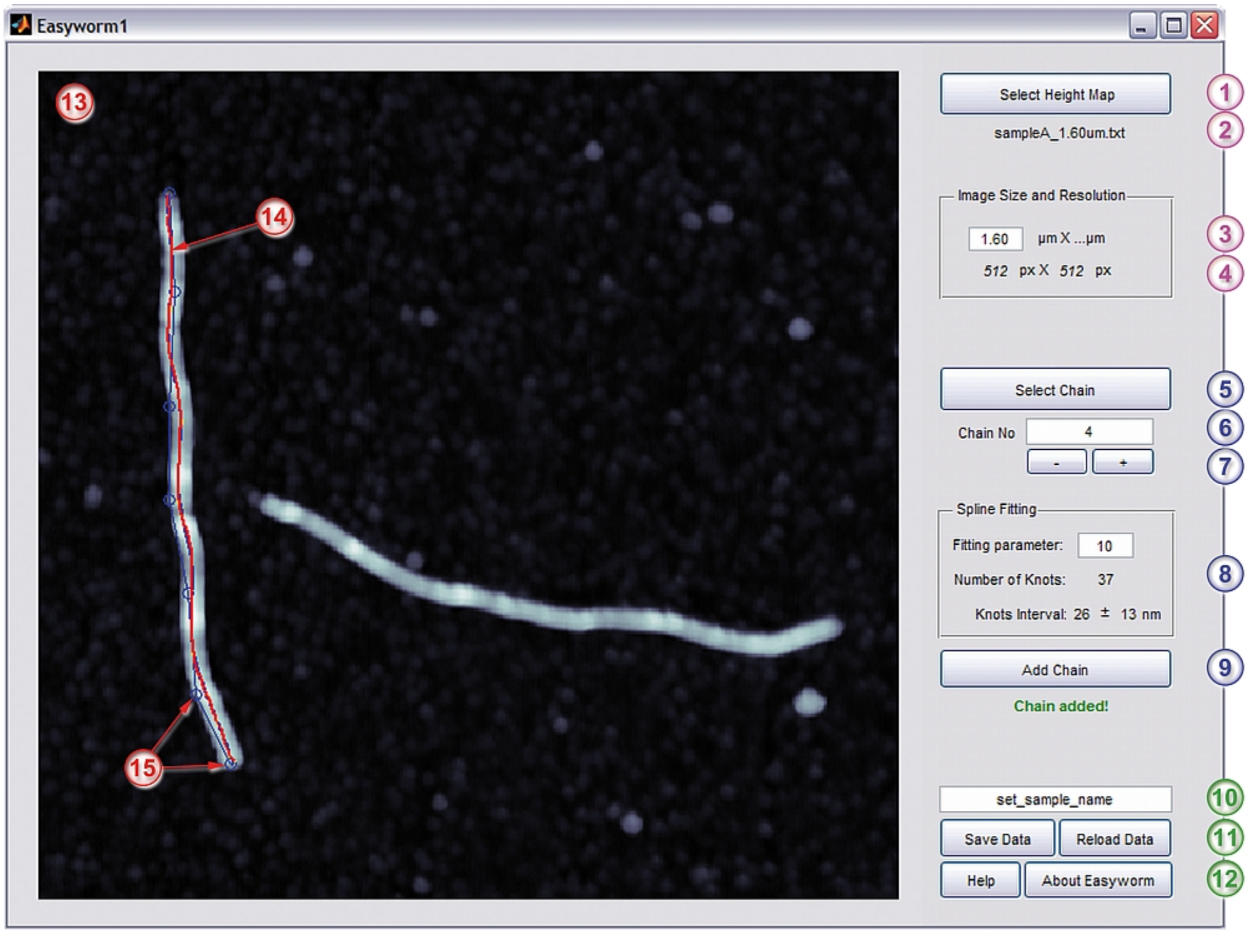

Figure S1: First graphical user interface (GUI) of the Easyworm software suite, called Easyworm1. After loading a height map, the Easyworm1 GUI allows fitting the contour of polymer chains to parametric splines (red lines; see Note S1). Eventually the data collected for all chains will be saved in one single .mat file in order to be loaded in the second GUI Easyworm2 for analysis (Fig. S2).

Figure S2:

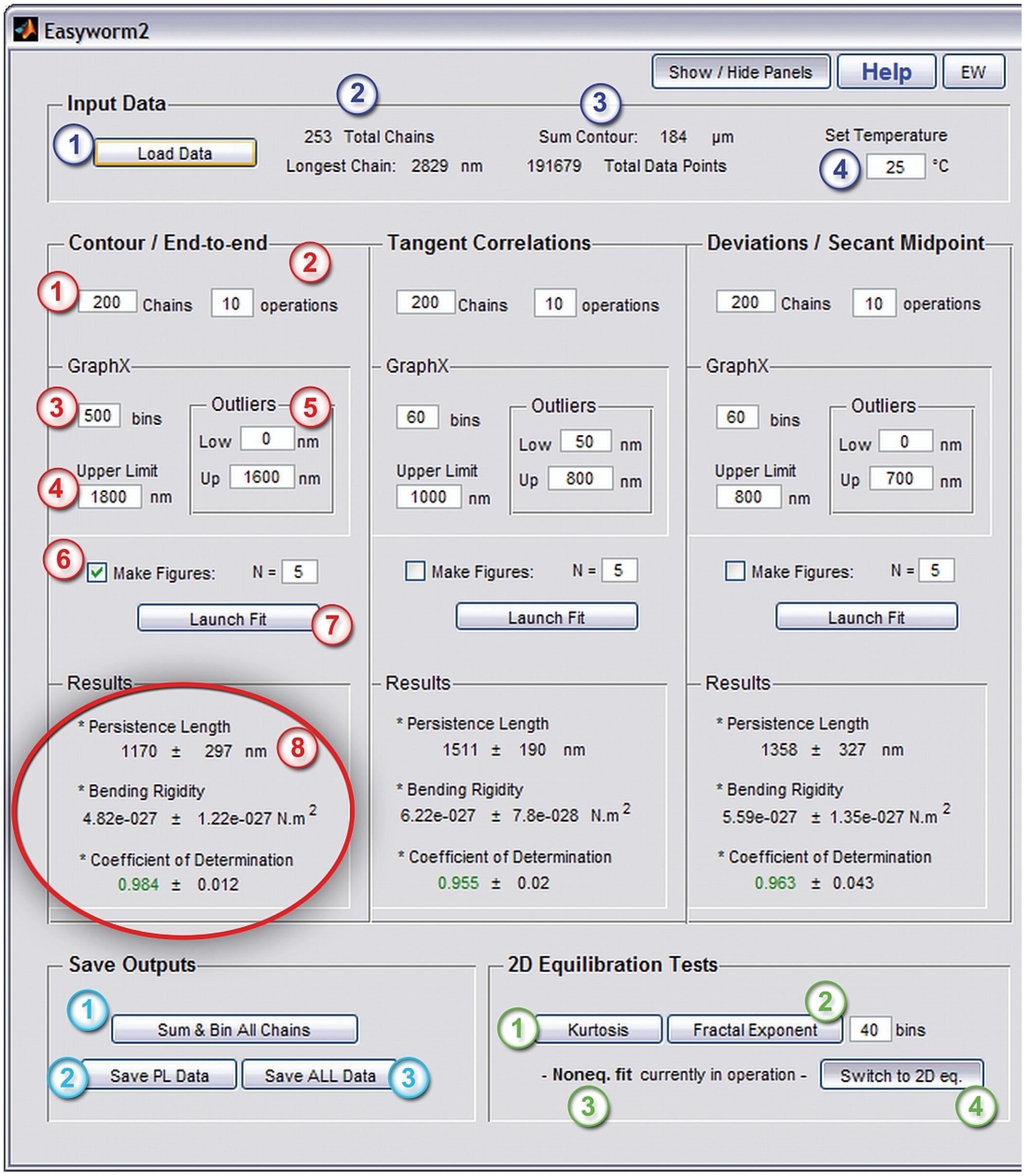

Figure S2a: Second graphical user interface of the Easyworm software suite, called Easyworm2 (left side). Easyworm2 provides a full set of complementary analysis tools that can be used on the .mat file generated by Easyworm1. Easyworm2 allows the determination of the persistence length P, provides statistical information on the contour length of the polymers, and contains a function that plots the fibrils with the initial tangents aligned. It can also test whether or not the polymer chains have equilibrated in 2D, and calculate the axial elastic moduli of the polymers according to diverse models. All details of the various functions of Easyworm2 and all the instructions how to use it are given in the Note S2.

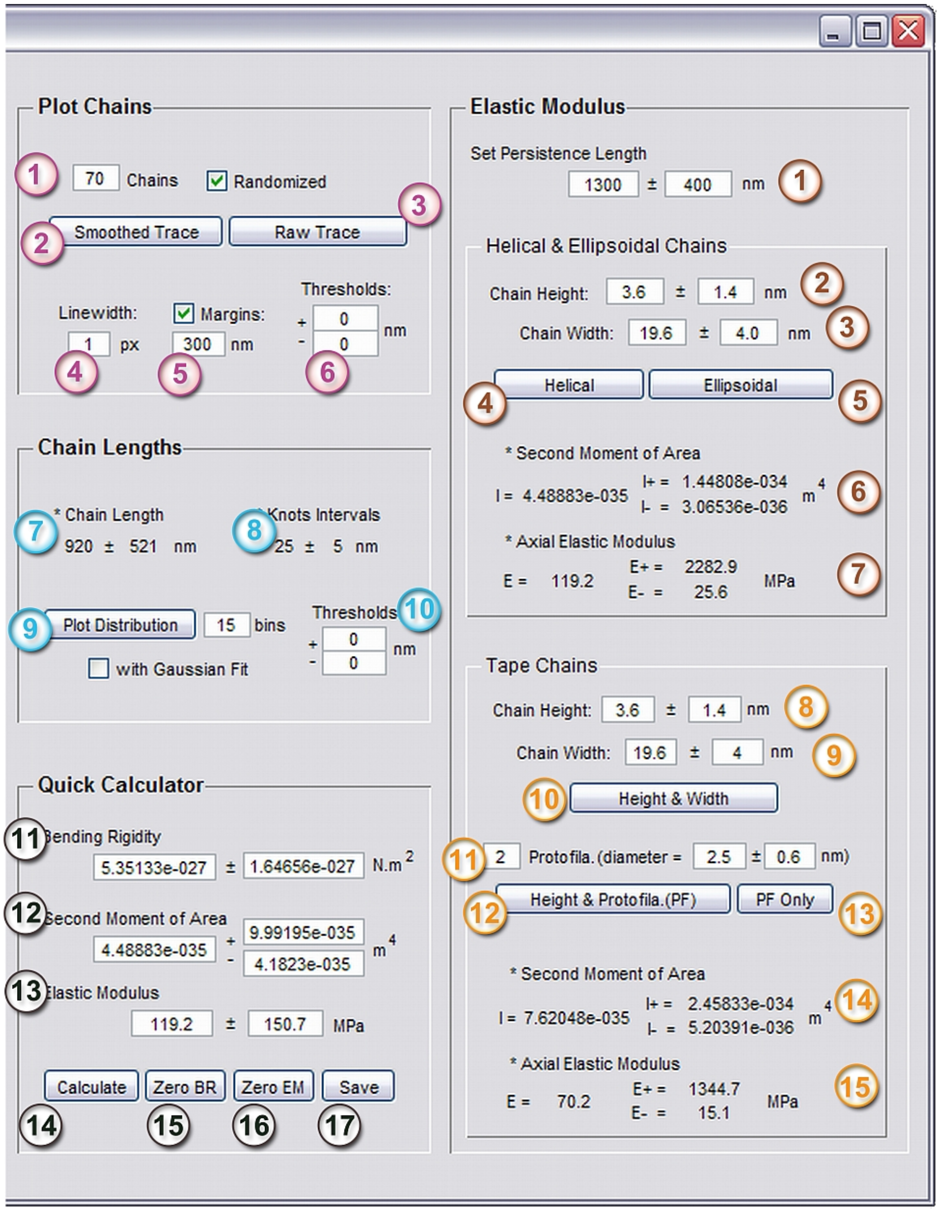

Figure S2b: Second graphical user interface of the Easyworm software suite, called Easyworm2 (right side).

Figure S3:

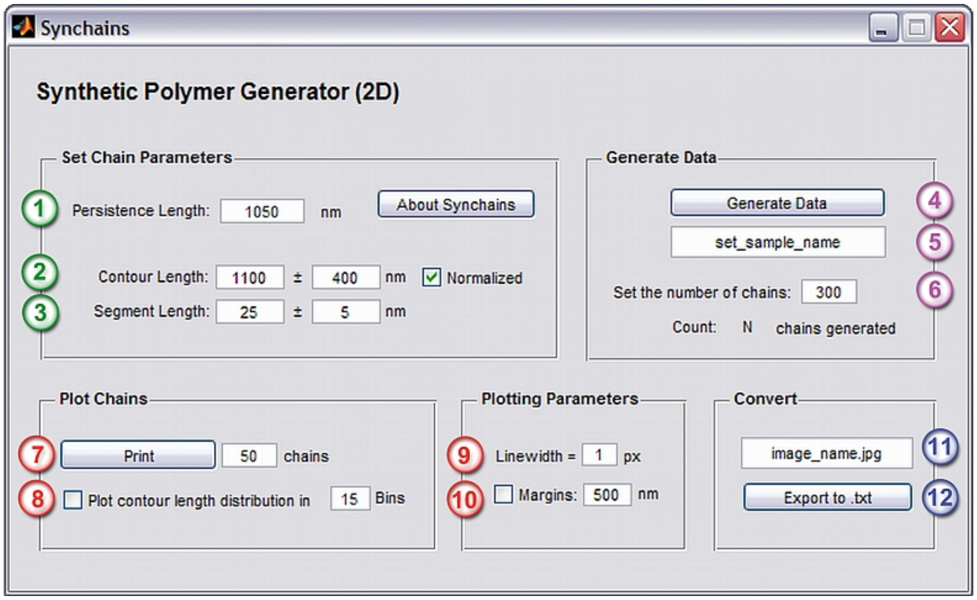

Figure S3: Graphical user interface to generate synthetic polymers, called Synchains. Instructions to use Synchains are given in the Note S3.

Figure S4:

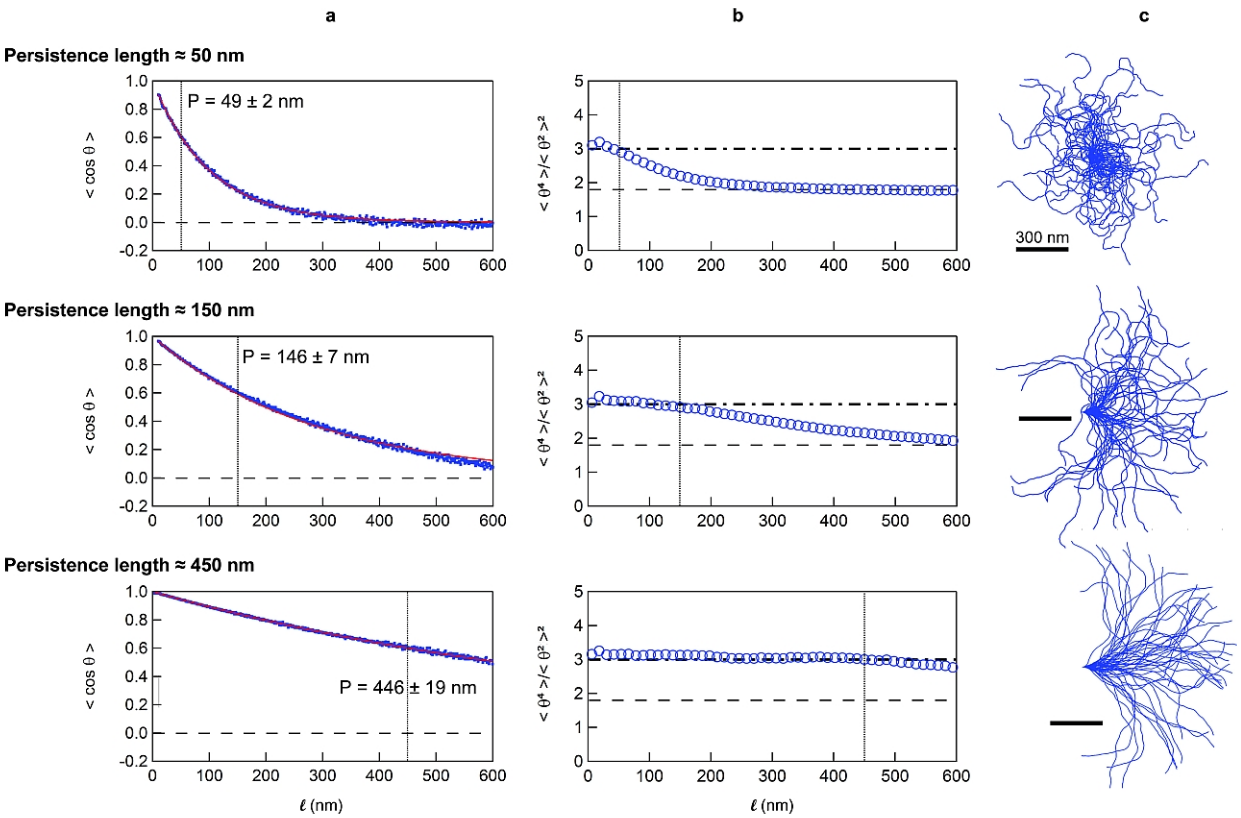

Figure S4: Correlation between the persistence length and the decay of the kurtosis of the θ distribution in synthetic 2D-chains. Three distinct *in silico* samples, with persistence lengths P of 50, 150, and 450 nm, were generated using Synchains (see Notes S3 and S4). **a**, cos θ as a function of ℓ . ℓ is the distance between two short chain segments forming an angle θ. Worm-like chain model fitting curves to data (see Eq. 1) are shown as red lines. The persistence lengths P derived from these fits are indicated in the graphs. **b**, Kurtosis of the θ distribution as a function of ℓ . The kurtosis is the ratio of the even moments of the distribution (see Eq. 4). The kurtosis is very close to the theoretical value of 3 (dash-dot line) for values of ℓ lower than P. It indicates that the θ distribution is Gaussian, thereby reflecting the fluctuations of fibril shape in a 2D-space. **a**,**b**, Vertical lines indicate the value of P set for each sample when their corresponding chains were generated. Dashed lines at cos θ = 0 (**a**) correspond to the dashed lines in **b** where the kurtoses are equal to 1.8 (*i.e.* the θ distribution is uniform between –π and + π). **c**, Synthetic chains (analyzed in **a** and **b**) plotted with their initial tangents aligned to facilitate visualization of shape fluctuations.

Figure S5:

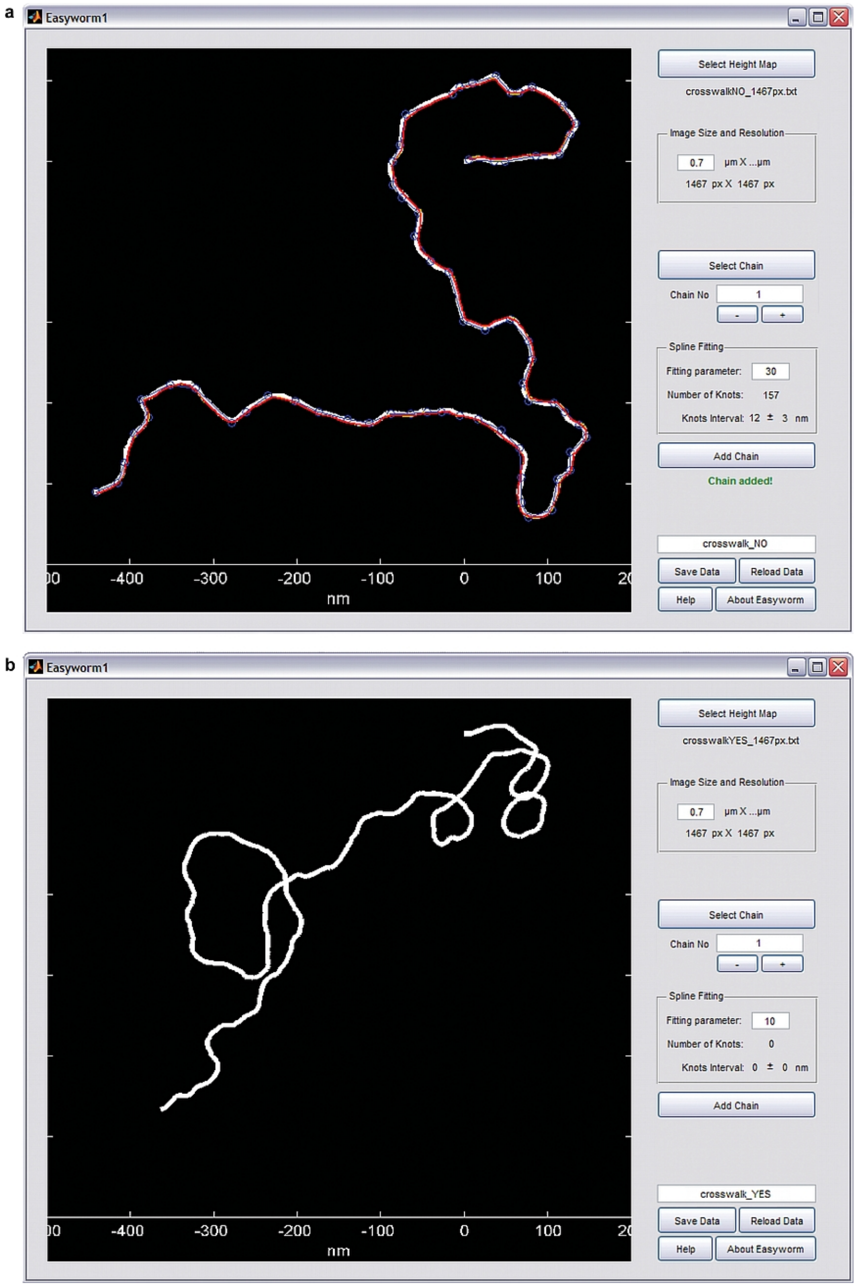

Figure S5: Synthetic polymers analyzed with Easyworm1. **a**, Two dimensional (2D)-chain displaying self-avoiding random walk. The red line is a fit of the chain contour to a parametric spline, as in Fig. S1. **b**, Chain displaying non-self-avoiding random walk in 2D (due to random angles between chain segments, the chain sometimes intersects its own path). All the chains similar to the one displayed in **b** were excluded from the analysis of the SP50 sample (see Tables 1 and S1), thereby giving rise to an increase of ~20 nm in the apparent persistence length (from 50 to ~70 nm). A similar increase was observed in previous studies and explained by excluded volume effects [5] (corresponding to a self-avoiding random walk in 3D) due to repulsive interactions between different parts of the same chain.

Note S1: Step-by-step instructions to fit the contour of chains to parametric splines using Easyworm1.

Preliminary Note: Pressing the *Help* button (⑫) will display a quick guide to get started with Easyworm1.

1. **1.** Prepare your height map either under the form of an image file of $n \times n$ pixels, or under the form of an $n \times n$ matrix embedded in a .txt file.

   Note: In case you load an image file, make sure that it is uniform in terms of color code, *i.e.* use an image coded in grayscale or a color image displaying variations of shades of the same color. The height map is generated according to the brightness of the pixels, with the brightest pixels corresponding to the highest peaks.

   Note: A minimum resolution of 256 × 256 pixels is necessary to carry out reliable measurements, although it will probably be insufficient if the chains analyzed look very small (and thin) on the image. In the latter case, using images with 512 × 512 pixels or even higher is recommended.

   In case you want to load a text file, each column should be separated by a tab delimiter. The $n \times n$ elements correspond to the $n \times n$ pixels of the image, where each specific n has a value corresponding to a specific height. Ideally, the name of the .txt file should indicate the size of the image (②).

   Note: The height map must be located in the same folder than the **Easyworm1** executable.

2. **2.** Pushing on button ① (*Select Height Map*) will pop-up a window asking for the height map to be loaded.

   Note: If it is an image file, it must be one coded in the formats that correspond to the following extensions: .bmp, .png, .jpg, .ppm, .tif.

   The height map can be an atomic force microscopy (AFM) image but not necessarily. It can also be an image taken by any other type of microscope, such as electron, contrast phase or even fluorescence microscope. Once properly loaded, the height map appears (⑬) and, at the same time, the name of the file is displayed in ②, and its resolution in ④.

3. **3.** Enter the size of the image (③).

4. **4.** Click on *Select Chain* (button ⑤). A gun sight will appear. Move it with your mouse at one extremity of the polymer chain and do a left-click on the mouse. Using the same procedure, manually add as many points as you want along the chain contour, up until you reach the other extremity. Then press Enter (↵) on the keyboard of your computer.

5. **5.** As a result of Step **4**, a red line that is a parametric spline fit to the chain contour should be displayed (⑭). If you are not satisfied with the fit, click on *Select Chain* (⑤) again and repeat Step **4**. If you are satisfied with the fit, note that some statistical information about the fit is now displayed (⑧).

Note: At this stage it is important to draw the user's attention on how the fit is done exactly. When "Enter" is pressed at Step **4**, the code will perform a series of operations that consist in looking at the highest points of the map between two points defined by the user. User input points are marked by blue circles (⑮), and the path found by the code is marked by a thin yellow line (not visible in Figure S1, as the red line is superimposed on top of it). The red line (⑭) is the result of a least-square fit of the initial path found by the code (*i.e.* the thin yellow line). The code will then store the red line as a finite set of points with specific coordinates that correspond to the knots of a parametric spline.

6. Modify the fitting parameter (⑧) if you want to increase or to decrease the number of knots associated with the spline representing one given chain. In that case, you will also need to repeat Step **4**.

Note: It is recommended to lower this parameter as much as possible in order to increase the number of data points to be used thereafter for analysis purposes. However, setting this parameter at a value that is too low could result in a distortion of the fit, and even in an increase of computational time needed to perform the calculations that come next.

7. Once you are satisfied with the fit shape and spline parameters, press button ⑨ (*Add Chain*). Depending on your computer speed, on how long the chain is and on how many knots the fitted spline is made of, it might take up to a few seconds to complete. Once it is, the number in textbox ⑥ will automatically be supplemented by 1 unit.

8. Go back to step **4** and repeat to add other chains.

Note: If you made a mistake, realize it later on (*e.g.* 3 fibrils after you made it) and want to go back, simply use the minus button (⑦) to set the chain to the number of your choice. The new data will overwrite the old data.

9. Go back to step **1** and repeat if you have additional height maps.

10. When all the chains have been analyzed, set your sample name in textbox ⑩.

Note: You will not be prompted to choose a filename; this will be your chosen sample name.

11. Use *Save Data* button (⑪) to generate the file that will compile all the data generated for each fibril in one single .mat file that is analyzable by **Easyworm2** (Fig. 2). The generated .mat file can be found in the folder where the **Easyworm1** executable is located.

Note: At this stage, **Easyworm1** can be closed and re-opened at a later date to reload the .mat file that has just been generated (by pressing *Reload Data*, ⑪), in case some supplementary chains of the same sample need to be added. In this case the chain number in textbox ⑥ will automatically be re-set appropriately.

Note S2: Step-by-step instructions to analyze the data using Easyworm2.

Preliminary Note: Pressing the **Help** button (located at the top of the interface) will display a quick guide to get started with **Easyworm2**.

- **Input Data panel** (Figure S2a)

  1. Load the .mat file that contains the data generated either by **Easyworm1** (see Fig. S1 and Note S1) or by **Synchains** (see Fig. S3 and Note S3) by pushing button ① (*Load Data*).

     Note: Once the data is properly loaded, some general information on the sample is displayed, both in the **Input Data** panel and in the **Chain Lengths** panel (see further in this Note). This information consists in: the total number of chains contained in the sample and the greatest contour length of the sample (②), as well as the sum of all contour lengths of all chains, and the total number of data points available for subsequent fitting procedures (③).

  2. Set the temperature in textbox ④. It should be the temperature of the buffer you used when the polymer chains have been immobilized on their substrate. By default it is set to 25°C (*i.e.* 273.15°K).

- **Contour / End-to-end panel** (Figure S2a)

  3. Press the *Launch Fit* button (⑦). At this stage it is recommended to push the same button in **Tangent Correlation** and in **Deviations / Secant Midpoint** panels. After this operation that corresponds to initializing the fitting parameters, the *Upper Limit* (④) as well as the upper fit edge (⑤) will be updated to the maximum value available. Results will be displayed in the *Results* subpanel, (⑧) but they should not be considered at this stage.
  4. Refine the fitting by optimizing all the parameters, after which the *Launch Fit* button (⑦) should be pressed again. It is recommended to check the box *Make Figures* (⑥) during that step. The number of bins (③) should be optimized along with the *Upper Limit* of the fit (④). The fitting quality can be estimated by the *Coefficient of Determination* (⑧) calculated for each fit. The number of resampling *operations* (see Methods), set by the number in textbox ②, should not deeply affect the final result, but a too great value could be computationally

expensive. The number of figures and fits displayed can only be lower or equal to the value set in ②.

Note: You can change the number of chains on which you choose to perform the analysis in textbox ①. After clicking the *Launch Fit* button (⑦), it will also update the information displayed in the **Input Data** and **Chain Lengths** panels.

Note: It is possible to exclude bins from the fit by setting upper and lower boundaries in panel ⑤. All bins lower and greater, respectively, than the values entered in *Low* and *Up* textboxes will then be considered as outliers. However, make sure that the *Up* textbox (in ⑤) is set to a value lower than that in the *Upper Limit* textbox (④) or it may not work properly.

5. Now that you know approximately what the persistence length of your sample is, it is time to check whether or not your fibrils have fully equilibrated in 2D.

- **2D Equilibration Tests panel** (Figure S2a)

6. Push on the *Kurtosis* button (①). A graph looking like those in Fig. S4b will pop up. If the blue markers in the figure stay close to 3 up until the value of the persistence length, then consider that your polymer chains have fully equilibrated on the substrate where they were seeded. In this case, do not touch anything (2D fit is the default value). If for any reason you already have pushed on toggle button ④ then click on it again until the text in ③ reads: - ***2D eq. fit*** *currently in operation* -. It corresponds to setting the s parameter in Eqs. 1–3 to a value of s = 2.

7. If the kurtosis does not equal 3, then you can consider that the polymers have not fully equilibrated on the 2D surface. This happens when only some parts of the polymer chain touch the surface and remain attached, due to strong interactions with the substrate. In this case, the non-equilibrated state is approximated by calculating the midpoint fluctuations between 2D and 3D states [2], which corresponds to setting the s parameter to a value of s = 1.5. In order to fit your data (by clicking *Launch Fit* buttons) using this setting, all you need to do is pushing on toggle button ④ until the text in ③ reads: - ***Noneq. fit*** *currently in operation* -.

Note: If the kurtosis value is not convincing enough to decide whether you should use one type of fit or the other, another 2D test is available by pressing the button ②. It calculates the mean end-to-end distance as a function of the contour length, and performs a linear regression analysis within the boundaries fixed in the **Outliers** subpanel of the **Contour / End-to-end** panel. If the slope of the linear fit is equal to ¾ for values of

the contour length higher than the persistence length, then it means that the polymer chains have fully equilibrated in 2D [6]. However, be cautious when using this function and use it only if the contour length of your chains is higher than their persistence length.

- **Tangent Correlations and Deviations / Secant Midpoint panel** (Figure S2a)

8. Steps **3–5** can be repeated for both of these panels. A graphical guide indicating which method should be used (depending mostly on the persistence length of the sample considered) is provided in Fig. 4. Because the measure to fit the data in the **Contour / End-to-end** panel is a direct derivation of the measure used in the **Tangent Correlations** panel, it is highly probable that they will return similar results whatever the characteristics of your sample are. In any case it is recommended to choose the results from the measure that returns the highest coefficient of determination.

<u>Note:</u> Use the **Deviations / Secant Midpoint** panel only if the persistence length P is much higher than the contour length L of your chains, *i.e.* L << P. Otherwise it will lead to systematic errors.

- **Save Outputs panel** (Figure S2a)

9. If you have used **Easyworm2** only to calculate the persistence length of a polymer sample, clicking on *Save PL Data* (button ②, where PL means the persistence length) will gather all relevant information and print them in .txt file outputs.

<u>Note:</u> It is *essential* to have clicked at least once on the *Sum & Bin All Fibrils* button (①) and on the *Test 2D* button (in the **Surface Parameter** panel) prior to pressing *Save PL Data*. Otherwise, the functions associated with the use of button ① will not compile properly and the output file will not be created.

10. Pressing button ① compiles and bins the data for all the fibrils, according to each method, instead of compiling data for only N/2 chains (*i.e.* randomly selected subset; see Step **4** and Methods, "Uncertainties on persistence length calculations" section), where N is the total number of chains available for analysis. The results of this compilation is stored and sent to text outputs when *Save PL Data* (button ②) is pressed. Note that if the *Make Figures* checkboxes are checked in the above panels, corresponding figures will be displayed. The number of bins in each of these figures is the value set in each *GraphX* subpanels.

11. If you have not only persistence length data but also generated data with the **Elastic Modulus** panel (see further in this note), press the *Save ALL Data* button (③). It will print .txt file outputs containing all the results generated during the analysis.

- **Plot Chains panel** (Figure S2b)

12. Use the *Smoothed Trace* (②) or *Raw Trace* (③) buttons to plot *n* chains (set the number in textbox ①) with their initial tangents aligned (in order to facilitate visualisation of the shape fluctuations according to the value of the persistence length; see Fig. 1c and Fig. S4).

Note: *Raw Trace* plots the chains with the fitting parameter set by the user for each chain in **Easyworm1** (see Fig. S1 and Note S1). *Smoothed Trace* plots the chains with the fitting parameter automatically set to 4 times the initial fitting parameter set by user. Also note that some margin can be added; check box (⑤) to add blank spaces between fibril extremities and the axes of the graphs displaying chains. The minimum distance of those blank spaces can be set in textbox ⑤.

13. Optional: check the *randomized* box next to textbox ① if you wish to randomize the fibrils selected for plotting and set the number of fibrils that should be plotted. The width of the splines plotted can be set in textbox ④. Finally, chains can be excluded from the plot if upper or lower thresholds are set in textboxes ⑥ (no threshold will be applied if the "+" textbox is set to 0).

- **Chain Lengths panel** (Figure S2b)

14. Use *Plot Distribution* button (⑨) to plot a histogram of the contour length distribution. Checking the "*with Gaussian Fit"* box will plot a histogram and fit a Gaussian to the distribution. The number of bins and threshold lengths (⑩) can be set as desired (no threshold will be applied if the "+" textbox is set to 0).

Note: The fibril length displayed in ⑦ is the Mean ± Standard Deviation (SD) calculated over all the chains of the sample. It is shown right after loading the .mat file. Some information on the interval between spline knots resulting from the fitting made with **Easyworm1** is exhibited in ⑧. For each Spline S the Mean(S) ± SD(S) of the interval between two consecutive spline knots is recorded (see Fig. S1). Then the Mean ± SD of all Means (over all of the splines of the sample) is calculated when the .mat file is loaded in the Easyworm2. It is displayed as Mean(Means) ± SD(Means) in ⑧.

- **Quick Calculator panel** (Figure S2b)

15. This panel can be used to convert values of bending rigidity (⑪) and second moment of area (⑫) into an elastic modulus (⑬) according to Eq. S1 (see Methods). Push on the *Calculate* button (⑭) when two out of the three parameters have been given a nonzero value. Zeroing the bending rigidity (by pushing on *Zero BR*; ⑮) or the elastic modulus (by pushing on *Zero EM*; ⑯) will switch the functionality of the *Calculate* button, by calculating one or the other. The uncertainties are calculated according to the propagation of the uncertainties of each variable. Clicking on the *Save* button (⑰) will generate a .txt file output.

- **Elastic Modulus panel** (Figure S2b)

16. Enter the persistence length (①) determined through Steps **3–7**.

17. In the *Helical and Ellipsoidal Chains* subpanel, enter the diameter of the polymer chain (②). It corresponds to the chain height that is measured by atomic force microscopy.

18. Press the *Helical* button (④) or the *Ellipsoidal* one (⑤) to calculate the cross-sectional second moment of area I of the polymer (see Methods).

<u>Note:</u> If your polymer can be modelled by a rod with a circular cross-section, leave a 0 value in the width textbox (③). The program will calculate I, I_+ and I_- using Eq. S2 (see Methods) after pressing the *Helical* button. If the width of your polymer is different than its height, and if you have a reliable measurement for it (*e.g.* from electron microscopy images), enter it and click on the *Ellipsoidal* button (⑤). The program will calculate I using Eq. S3, that is, considering an ellipsoidal cross-section.

19. After clicking the *Helical* or the *Ellipsoidal* buttons, I, I_+ and I_- are displayed in ⑥. If a nonzero persistence length has been entered in ①, the software will automatically calculate and display elastic moduli E, E_+ and E_- (⑦), using values determined for I, I_+ and I_- (⑥), respectively. In the meantime, it will update the values in the **Quick Calculator** panel.

<u>Note:</u> The positive and negative uncertainties of I are not symmetric. Importantly, also note that the **Quick Calculator** panel displays the uncertainties ΔI_+ and ΔI_-, while the *Helical and Ellipsoidal Chains* subpanel displays I_+ and I_-, respectively equal to $(I + \Delta I_+)$ and $(I - \Delta I_+)$.

20. Use the subpanel *Ribbon / Tape Chains* to calculate I according to a rectangular cross-section, as in Eq. S4 (see Methods). Three distinct ways of determining I_R are available:

- Set the fibril height (⑧) and width (⑨) and press *Height & Width* (⑩). This is the most straightforward manner if both height and widths measurements are available. This method does not consider any number that might be set in the three textboxes related to protofilaments sizes (same line than label ⑪).
- Set the fibril height (⑧), the number of protofilaments n_f in the mature fibril (⑪) and the diameter of a single filament ϕ_f (which cross-section is assumed to be circular). Pressing *Height & Protofilaments (PF)* (⑫) will calculate I_R by assuming a width equal to $n_f \times \phi_f$. This method does not consider any number that might be set in textbox ⑨.
- Set n_f and ϕ_f only. Then press the *PF Only* button (⑬). I_R will be calculated as in the previous method, except for the height that will be taken equal to ϕ_f. This method does not consider any number that might be set in textboxes ⑧ and ⑨.

21. The results of the calculations made at Step **20** are displayed in ⑭ and ⑮. Remarks made about ⑥ and ⑦ at Step **19** also apply here.

Note S3: Step-by-step instructions to generate synthetic polymers using Synchains.

1. Set the persistence length of the synthetic chains (①).

2. Set the contour length (②) and the segment length (③) of the synthetic chains.

   Note: If a nonzero uncertainty is set to the segment length, it will correspond to the standard deviation of a normal distribution centered on the value set for the mean segment length. Check the box in ② to make the uncertainty set for the contour length correspond to the standard deviation of a normal distribution centered on the value set for the mean contour length. If the box is not checked, then the contour lengths generated will be random values picked in the interval comprised between $C_L - \Delta C_L$ and $C_L + \Delta C_L$, where C_L is the contour length and ΔC_L its uncertainty.

3. Set the number of chains that should be generated in textbox ⑥.

4. Clicking on the *Generate Data* button (④) will produce a .mat file similar to those that can be generated from experimental polymers using **Easyworm1**, and that can be analyzed similarly using **Easyworm2**. The name that the generated .mat file will have can be customized in textbox ⑤.

5. Clicking on *Print* (⑦) will simply generate a figure where random synthetic chains are displayed with their initial tangents aligned, as in the **Plot Chains** panel of **Easyworm2**. The number of chains plotted can be set in the textbox next to the *Print* button (⑦). Checking the box in ⑧ will produce another figure showing the distribution of the contour length of the fibrils generated. The number of bins in the histogram can be set in the textbox (⑧). The line width of the synthetic polymers can be set in textbox ⑨. Also note that some margins can be added at each side of the printed graph (⑩).

6. The button *Export to .txt* (⑫) can be used to convert an image file (only if it is generated by **Synchains**) to a .txt file that can be loaded in **Easyworm 1** (see Fig. S5). In this case, the image file must be in the same folder as the executable Synchains.exe, and its full name should be entered in textbox ⑪ (*e.g.* “image_name.jpg”). Also note that this works only when chains are generated one by one (set the number to 1 in textbox next to the *Print* button in ⑦). If you dispose of Matlab on your computer, you can also use the “synseries.m” script to generate multiple copies of such individual chains (the script is included in the software package).

Note S4: Details on how synthetic chains were generated and analyzed.

- On the synthetic chains in Fig. S4:

  Synthetic chains were generated with *Synchains* and analyzed using *Easyworm2* only in order to illustrate the variation of the kurtosis of the θ distribution as a function of P. We generated 3 distinct datasets of 500 chains. For each dataset, the contour length was set to 900 nm, ℓ segments to 10 ± 3 nm and P to 50 nm, 150 nm and 450 nm, respectively.

- On the synthetic chains in Tables 1 and S1 and in Fig. S5:

  In this *in silico* experiment, we used *Synchains* and both *Easyworm1* and *Easyworm2* to generate and analyze synthetic chains where P was varied from 50 nm (similar to the persistence length of DNA [5]) to 5.2 mm (similar to the persistence length of microtubules [1]).

  Precisely, synthetic chains were generated one by one and "materialized" as splines with a width of 3–5 pixels. Each image was then converted from a .tif to a .txt file containing an $n \times n$ matrix where $n \times n$ is the number of pixels in the image. Each n element was assigned the value of 0 for background, and of 255 for pixels representing the chain. Each .txt file was then loaded in Easyworm1 and analyzed like any other experimental test sample. The s parameter (see Eqs. 1–3) was set to a value of 2 accordingly to the intrinsic 2D-character of these chains.

Supplementary References

1. Gittes F, Mickey B, Nettleton J, Howard J: **Flexural rigidity of microtubules and actin-filaments measured from thermal fluctuations in shape.** *J Cell Biol* 1993, **120:**923-934.
2. Smith JF, Knowles TPJ, Dobson CM, MacPhee CE, Welland ME: **Characterization of the nanoscale properties of individual amyloid fibrils.** *Proc Natl Acad Sci U S A* 2006, **103:**15806-15811.
3. Lamour G, Yip CK, Li H, Gsponer J: **High Intrinsic Mechanical Flexibility of Mouse Prion Nanofibrils Revealed by Measurements of Axial and Radial Young's Moduli.** *ACS Nano* 2014, **8:**3851-3861.
4. Knowles TPJ, Fitzpatrick AW, Meehan S, Mott HR, Vendruscolo M, Dobson CM, Welland ME: **Role of intermolecular forces in defining material properties of protein nanofibrils.** *Science* 2007, **318:**1900-1903.
5. Rivetti C, Guthold M, Bustamante C: **Scanning force microscopy of DNA deposited onto mica: Equilibration versus kinetic trapping studied by statistical polymer chain analysis.** *J Mol Biol* 1996, **264:**919-932.
6. Valle F, Favre M, De Los Rios P, Rosa A, Dietler G: **Scaling exponents and probability distributions of DNA end-to-end distance.** *Phys Rev Lett* 2005, **95:**158105.
